# Studying Grain Boundary Strengthening by Dislocation-Based Strain Gradient Crystal Plasticity Coupled with a Multi-Phase-Field Model

**DOI:** 10.3390/ma12182977

**Published:** 2019-09-14

**Authors:** Waseem Amin, Muhammad Adil Ali, Napat Vajragupta, Alexander Hartmaier

**Affiliations:** 1 Interdisciplinary Center for Advanced Materials Simulation (ICAMS), Ruhr-Universität Bochum, Universitätsstr. 150, 44801 Bochum, Germany; Muhammad.Ali-u7x@rub.de (M.A.A.); napat.vajragupta@rub.de (N.V.); alexander.hartmaier@rub.de (A.H.); 2 Department of Metallurgy and Materials Engineering, University of Engineering and Technology, Taxila 47050, Pakistan

**Keywords:** phase field, crystal plasticity, Hall-Petch effect, dislocation density, micromechanics

## Abstract

One ambitious objective of Integrated Computational Materials Engineering (ICME) is to shorten the materials development cycle by using computational materials simulation techniques at different length scales. In this regard, the most important aspects are the prediction of the microstructural evolution during material processing and the understanding of the contributions of microstructural features to the mechanical response of the materials. One possible solution to such a challenge is to apply the Phase Field (PF) method because it can predict the microstructural evolution under the influence of different internal or external stimuli, including deformation. To accomplish this, it is necessary to take into account plasticity or, specifically, non-homogeneous plastic deformation, which is particularly important for investigating the size effects in materials emerging at the micron length scale. In this work, we present quasi-2D simulations of plastic deformation in a face centred cubic system using a finite strain formulation. Our model consists of dislocation-based strain gradient crystal plasticity implemented into a PF code. We apply this model to study the influence of grain size on the mechanical behavior of polycrystals, which includes dislocation storage and annihilation. Furthermore, the initial state of the material before deformation is also considered. The results show that a dislocation-based strain gradient crystal plasticity model can capture the Hall-Petch effect in many aspects. The model reproduced the correct functional dependence of the flow stress of the polycrystal on grain size without assigning any special properties to the grain boundaries. However, the predicted Hall-Petch coefficients are significantly smaller than those found typically in experiments. In any case, we found a good qualitative agreement between our findings and experimental results.

## 1. Introduction

The properties of engineering materials are size-dependent if the microstructural length scale falls into an order of a few microns to less than a micron [1,2]. The pioneering work of Hall and Petch [3,4] motivated many researchers to study the underlying physics and the influence of the grain size effect on the mechanical behavior of materials [5,6,7,8,9,10]. Within the domain of metallic materials, the main plastic deformation mechanism is dislocation slip. This deformation mechanism depends on the density and evolution of the dislocations, crystal structures, and crystallographic orientations, and on the localization of deformation as a result of the gradients of the grain morphology and the distribution of grain sizes [11].

The grain size effect is the manifestation of the fact that a polycrystal with larger grains experiences larger strain incompatibility during plastic deformation. This generates higher internal stresses in the microstructure which leads to lowering of yield strength and as the grain size is reduced, an opposite phenomenon is observed [3,4]. For coarse grains, it is well understood that an increase of the dislocation density results in a strengthening of the microstructure, which can be described by Taylor’s hardening law [12]. In the case of fine or ultra-fine grained materials, their strength is grain size dependent [2,5,13,14]. Furthermore, this is associated with the state of the material, e.g., its initial dislocation density, which determines the strength of a material [15].

Because of a significant improvement in computational power in recent decades, the mechanical behavior of crystals has been simulated extensively by using crystal plasticity (CP) models. Such plasticity models relate the evolution of the plastic flow of a crystal as a result of its state and the evolution of this state [11]. However, classical CP models do not include any intrinsic microstructural length scale and therefore fail to describe the size dependent mechanical response of the materials [16]. This drawback can be coped with by employing strain-gradient CP models [17,18,19] that address size dependent plasticity. These models have proven their capability to describe the non-homogeneous deformation by taking into account the plastic strain at any material point and its influence on the neighbouring points. This involvement of the plastic strain gradient can therefore capture the grain size effect [17], and such models can also be formulated on the basis of dislocation mechanics. To serve this purpose, the dislocations can be divided into two relevant categories: (1) statistically stored dislocations (SSD) and (2) geometrically necessary dislocations (GND). The arbitrary dislocation configurations occurring during plastic deformation generates SSD, whereas GND emerge from sites of non-homogeneous deformation, mainly at the interfacial regions [20,21]. One main characteristic of SSD configurations is that their net Burgers vector is zero, whereas GND configurations possess a non-zero net Burgers vector. The evolution of SSD can be described on the basis of the Kocks-Mecking law [22], and GND can be evaluated on the basis of Nye’s dislocation tensor [23].

Although strain-gradient CP models are sensitive to microstructural features, they still lack the capability to describe the plastic deformation of a material in connection to the evolution of the microstructure during the processing steps [24]. This microstructural evolution is essentially related to the movement of interfaces or a changing chemical composition of materials. Such changes can numerically be tracked with the help of phase field models [25]. These models are very flexible and can incorporate certain physical phenomena of interest by including properly defined energy densities into the description of the total energy of the system. Therefore, the characteristics of these two types of models can be superimposed to predict the mechanical response of materials along with their microstructural evolution.

Phase field models found numerous applications in materials science during the last decades, mainly to predict solidification dynamics [25]. The pioneering work of Khachaturyanet al. in the framework of phase-field microelasticity [26] set a new dimension of phase field modeling and enabled the development of phase field models to describe the elastic as well as the plastic deformation of materials. One variation of the PF method is the multi phase field model (MPF), which can predict the behavior of a system by incorporating an unlimited number of phase fields/physical quantities.

Some studies report how the phase field is coupled with isotropic plasticity [27] or CP [28] to analyze the finite or infinitesimal strains in larger material volumes. Recent studies also show an increasing trend towards discrete-dislocation-dynamics-based phase field models [29,30,31,32,33,34,35,36] to describe plastic deformation, but such models can only be applied to smaller systems due to the associated computational cost. The non-homogeneous deformation in the framework of phase field modeling has, however, been addressed by only a few researchers, who employed strain-gradient CP coupled with a phase field model like, for example, the work by Aldakheel on fracture analysis of metals [37]. Such an approach of coupling is very significant because it has paved the way to predict the response of complex microstructures under various boundary conditions. It can simultaneously track the microstructural evolution during material processing and the non-homogeneous deformation resulting from the external boundary conditions, which leads to the description of the mechanical properties of materials on the basis of their process history.

In our work, we present a dislocation-based strain-gradient CP coupled with the MPF model. One prominent advantage of the proposed framework is its capability to capture not only the material process history by tracking the evolution of the microstructure but to assess at the same time the dislocation structures to the level of finite strains as a result of plastic deformation.

The outline of this study is as follows. The second section comprises the model description including the MPF, deformation kinematics, and strain-gradient CP. The third section illustrates the simulation setup including the boundary conditions and the employed parameters. The fourth section presents and discusses the evolution of dislocation densities, the resulting flow stress and the governing mechanisms for the grain size effect. The fifth section discusses the results.

## 2. Model

The model used for our analysis consists of a MPF model as described in the Section 2.1. It involves the contribution of the elastic energy which is explained in the Section 2.2. This elastic energy is calculated with the help of the plastic strain predicted by a strain-gradient CP model, which is elaborated in the Section 2.3.

### 2.1. The Multi Phase Field Model

The MPF model followed in our work is the one developed by [38]. It can describe the microstructural evolution under the influence of different internal or external stimuli because of its strong interface tracking capability. It allows us not only to study a system with multiple components/phase fields including thermodynamic phases, chemical elements, number of grains, crystal orientations, and morphology, but also to address multi-physical phenomena simultaneously. A basic constraint, however, is that the summation of the magnitudes of all the individual phase fields $f_{\alpha }$ and $f_{\beta }$ should be equal to 1 in the respective bulks of the phases whereas the sum of the magnitudes of all the phase fields should be equal to the unity inside the interfacial region. Hence the value of each phase field $\phi_{\alpha }$ varies as $0⩽\phi_{\alpha }⩽1$ while traversing from the bulk of one phase field to the other phase field and given as

(1) $$\sum_{\alpha =1}^{N}\phi_{\alpha }\left(x\right)=1.$$

The evolution of the phase fields/microstructure is driven by the minimization of the total energy of the system. Therefore, an energy function is defined that can take into account all the energy densities of interest. It usually includes, but is not limited to, the energy contributions of the chemical, interfacial, and elastic aspects that lead to the evolution of a system. A general equation to describe the total energy content of a system is as follows

(2) $$F=\int_{\Omega }\;\left(f^{\mathrm{int}}+f^{\mathrm{el}}\right).$$

Here, F is defined as the energy functional to describe the state of the system, $f^{\mathrm{int}}$ is the interfacial energy density, and $f^{\mathrm{el}}$ is the elastic energy density. These quantities are integrated via the size of the domain Ω.

(3) $$f^{\mathrm{int}}=\sum_{\alpha =1,\beta >\alpha }^{N}\frac{8\sigma_{\alpha \beta }}{\eta }\left[-\frac{\eta^{2}}{\pi^{2}}\left(\nabla \phi_{\alpha }\;\cdot \nabla \phi_{\beta }\right)+\phi_{\alpha }\phi_{\beta }\right].$$

The interfacial energy density takes into account the interfacial thickness η and the energy $\sigma_{\alpha \beta }$ of the interface between the α and β phase/grain, which may be taken as isotropic or anisotropic. The interfacial width is chosen in such a way that it forms a diffused and stable interface during the evolution. The field variables or material properties at diffuse interfaces are evaluated by taking their linearly weighted average, more detail is provided in [39]. The elastic energy density is assumed to be a function of the elastic stiffness $C_{\alpha }$, total strain $\epsilon_{\alpha }$, eigenstrain (which is a stress-free strain) $\epsilon_{\alpha }^{*}$, and plastic strain $\epsilon_{\alpha }^{\left(p\right)}$ produced in each phase field $\phi_{\alpha }$ as given below

(4) $$f^{\mathrm{el}}=\frac{1}{2}\sum_{\alpha =1}^{N}\phi_{\alpha }\left[\epsilon_{\alpha }-\epsilon_{\alpha }^{*}-\epsilon_{\alpha }^{\left(p\right)}\right]C_{\alpha }\left[\epsilon_{\alpha }-\epsilon_{\alpha }^{*}-\epsilon_{\alpha }^{\left(p\right)}\right],$$

### 2.2. Elasticity

To describe finite strain, a generalized stress-strain relation is used:

(5) $$\sigma_{\alpha }=C_{\alpha }:\epsilon_{\alpha }^{\mathrm{el}}.$$

In this equation **C**${}_{\alpha }$ is the 4th order elastic stiffness tensor, $\sigma_{\alpha }$ is the 2nd Piola-Kirchhoff stress, “:” represents double contraction in the form (a${}_{ij}$):(b${}_{ij}$) = $\sum_{i}\sum_{j}$ a${}_{ij}$ b${}_{ji}$ and $\epsilon_{\alpha }^{\mathrm{el}}$ is the Lagrangian strain. Now, as the stiffness tensor and the elastic strains are known for each phase field, the evaluation of the driving force is simple. The continuum mechanical homogenization sets several rules and evaluates effective values of mechanical properties with the help of phase fraction and the parameters related to the phase. The resulting total strain ϵ should be weighted as the average of strains associated with a phase field as

(6) $$\epsilon =\sum_{\alpha =1}^{N}\phi_{\alpha }\left(\epsilon_{\alpha }^{\mathrm{el}}+\epsilon_{\alpha }^{*}+\epsilon_{\alpha }^{\left(p\right)}\right)=\sum_{\alpha =1}^{N}C_{\alpha }^{-1}:\sigma +\sum_{\alpha =1}^{N}\phi_{\alpha }\epsilon_{\alpha }^{*}+\sum_{\alpha =1}^{N}\phi_{\alpha }\epsilon_{\alpha }^{\left(p\right)}.$$

### 2.3. Plasticity

Plastic deformation is described in terms of plastic shear rate ${\dot{\gamma }}^{s}$ on a slip system s. It is calculated by using a dislocation-based strain-gradient CP model, which is taken from [40]. In this model, the plastic flow rule for a slip system is defined by Orowan’slaw. The shear strain rate of the slip system s is associated with the velocity $\nu^{s}$ and the total dislocation density $\rho_{\mathrm{total}}$, which is assumed to be the mobile dislocations on the same slip system and given as follows

(7) $${\dot{\gamma }}^{s}=\rho_{\mathrm{total}}^{s}b\nu^{s},$$

where b defines the magnitude of the Burgers vector. The dislocation slip velocity $\nu^{s}$ on the same slip system s is defined as

(8) $$\nu^{s}=\nu_{0}|\frac{\tau^{s}}{\tau_{c}^{s}}{|}^{\frac{1}{m}}.$$

Here, *m* describes the strain rate sensitivity of the material, and $\nu_{0}$ is the reference velocity of the dislocations, $\tau^{s}$ is the resolved shear stress along the slip system s, and $\tau_{c}^{s}$ is its critical value to start the dislocation slip, known as critical resolved shear stress (CRSS). It is defined through Taylor’s hardening law as

(9) $$\tau_{c}^{s}=\tau_{0}+c_{1}\mathrm{Gb}\sqrt{\rho_{\mathrm{total}}^{s}},$$

where $\tau_{0}$ is the lattice friction stress/static yield stress, c${}_{1}$ is a geometrical factor, and *G* is the shear modulus. $\rho_{\mathrm{total}}^{s}$ is a measure of the total dislocation content of the slip system s, in our study it consists of SSD and GND, as follows

(10) $$\rho_{\mathrm{total}}^{s}=\rho_{\mathrm{SSD}}^{s}+\rho_{\mathrm{GND}}^{s}.$$

The magnitude of this total dislocation density in Equation (9) at the initial state is $\rho_{\mathrm{total}(i)}$ and it is assumed as equivalent to the initial magnitude of $\rho_{\mathrm{SSD}}^{s}$. The evolution of SSD is based on the Kocks-Meckinglaw:

(11) $${\dot{\rho }}_{\mathrm{SSD}}^{s}=(k_{1}\sqrt{\rho_{\mathrm{SSD}}^{s}+\rho_{\mathrm{GND}}^{s}}-k_{2}\rho_{\mathrm{SSD}}^{s}){\dot{\gamma }}^{s}.$$

where k${}_{1}$ is a measure of storage of SSD, while k${}_{2}$ is the measure of annihilation of SSD. Plastic strain is summation of the product of shear strain $\dot{\gamma }$ and the symmetric part of the Schmidt tensor $P^{s}$ for every s slip system. Schmidt tensor is described through the direction vector of dislocation slip $d^{s}$ and the vector of the slip plane normal $n^{s}$. Thus, evolution of plastic strain is given by

(12) $${\dot{\epsilon }}^{\left(p\right)}=\sum_{s=1}^{N}{\dot{\gamma }}^{s}P^{s}\;,\;P^{s}=\frac{1}{2}\left(d^{s}⊗n^{s}+n^{s}⊗d^{s}\right).$$

The resulting plastic strain is then used in Equation (4) to determine the contribution of the system’s elastic energy and to predict the concurrent microstructural evolution. The gradient of the evolution of this plastic strain defines the evolution of the Nye’s dislocation tensor described by

(13) $$\dot{\Lambda }={\left(-e_{\mathrm{jkl}}{\dot{\epsilon }}_{\mathrm{il},k}^{\left(p\right)}\right)}^{T}e_{i}⊗e_{j},$$

where $-e_{\mathrm{jkl}}$ is the third order permutation tensor(having a value of 1 with even permutation of the indices, −1 with odd permutation order and 0 otherwise), ${\dot{\epsilon }}_{\mathrm{il},k}^{\left(p\right)}$ defines the partial derivative of the plastic strain rate with respect to the coordinate k, such that $e_{\mathrm{jkl}}$${\dot{\epsilon }}_{\mathrm{il},k}^{\left(p\right)}$ is the rotation of the plastic strain rate, and ⊗ represents the diadic product between the Cartesian $e_{i}$ and $e_{j}$ unit vectors, which define the components of the resulting tensor. The evolution of GND can now be described as follows

(14) $${\dot{\rho }}_{\mathrm{GND}}^{s}=\frac{1}{b}\left(\right|d^{s}\dot{\Lambda }l^{s}\left|+\right|d^{s}\dot{\Lambda }d^{s}\left|\right).$$

Here, **d** and **l** refer to the slip direction vector and to the line direction vector used to evaluate the edge (first term in this equation, **l** is normal to **d**) and screw components (second term, where **l** is parallel to **d**) of GND. These vectors are given in Table 1 for each slip system.

## 3. Simulation Setup

The dislocation-based strain-gradient CP model is implemented with an explicit integration scheme into the open source phase field code OpenPhase [41]. The mechanical problems in this context are solved by using the spectral elastic solver [28], which maintains the mechanical equilibrium on the basis of the Saint-Venanthyperelastic material model [42]. This material model extends the typical linear elasticity to the nonlinear regime and relates the Lagrangian strain with the 2nd Piola-Kirchoff stress. In the present study, we focus on the Face-Centred Cubic (FCC) system, and we consider the dislocation glide on the crystallographic slip systems {111}〈110〉. Furthermore, we assume the total dislocation density to be equivalent to SSD as well as to the mobile dislocation density at the start of the simulations. The material parameters that we apply are mostly taken from the literature [20,40,43,44] and are summarized in Table 2.

**Figure 1:**
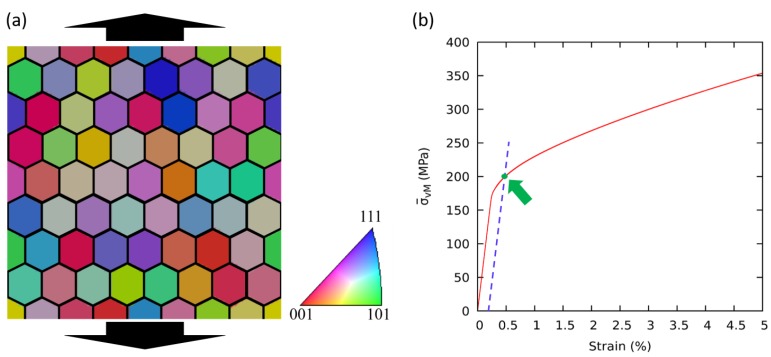
(**a**) Orientation distribution, (**b**) flow stress for a polycrystal with grain diameter of 0.4 μm and an offset of 0.2% of global plastic strain to define the onset of plastic yielding

To perform micromechanical simulations, quasi-2D periodic Representative Volume Elements (RVE) consisting of 64 grains with a regular hexagonal shape, are generated with the general Voronoi tessellation by embedding the tessellation module of the Voro++ library [45] into OpenPhase. For the sake of computational efficiency, the number of grains has been kept 64 with a honeycomb structure consisting of eight rows and eight columns. The number of grains is reasonable to obtain good statistics for this study. Furthermore, a similar honeycomb structure with similar random crystallographic orientation sets is used for all RVEs to avoid any unwanted influences, which can affect simulation results. Four RVEs with grain diameters of 16, 1.6, 0.8 and 0.4 μm are created to investigate the influence of the grain size on the mechanical response of the material. To exclude the influence of texture on the deformation behavior, similar sets of random crystallographic orientations are assigned to all RVEs, resulting into a microstructural texture index value close to 1, this index is a measure of the randomness of crystal orientations and the value 1 refers to completely random orientations i.e., absence of any texture. The geometry of an RVE used in this study is shown in Figure 1a. The color of each grain corresponds to the color code of the Inverse Pole Figure (IPF), usually evaluated by an Electron Backscatter Diffraction (EBSD) analysis, and black arrows denote the loading direction.

To solve the phase field evolution, interfaces between the crystals/grains are diffused for obtaining a certain interfacial thickness η. The interfacial energy $\sigma_{\alpha \beta }$ is assumed to be isotropic in order to prevail any effect of interfacial anisotropy and subsequent influence on the grain size effect. The periodic boundary conditions are applied to all of the phase fields along the regular computational grid. Same boundary condition is applied in the thickness direction of RVE. Isothermal and uniaxial tensile strain is applied at room temperature as loading condition with a constant strain rate of 0.1 s${}^{-1}$ to produce a total deformation of 5%. Grain growth is restricted by assuming very low interfacial mobility.

(15) $$|\epsilon^{\left(p\right)}|=\sqrt{\left(\epsilon_{1}^{2}+\epsilon_{2}^{2}+\epsilon_{3}^{2}+\frac{1}{2}\epsilon_{4}^{2}+\frac{1}{2}\epsilon_{5}^{2}+\frac{1}{2}\epsilon_{6}^{2}\right)}$$

The flow stress is homogenized by taking the volume average of the von Mises equivalent stress $\sigma_{\mathrm{vM}}$, whereas the equivalent plastic strain $\epsilon^{\left(p\right)}$ is calculated by the Frobenius norm [46] as given in Equation (15), in which $\epsilon_{1}$, $\epsilon_{2}$, $\epsilon_{3}$ represent the normal strains and $\epsilon_{4}$, $\epsilon_{5}$, $\epsilon_{6}$ represent the shear strains of the strain tensor in Voigt notation. Yield strength is calculated by taking an offset of the elastic part of the stress-strain curves at 0.2% of the total strain as shown in Figure 1b.

**Figure 2:**
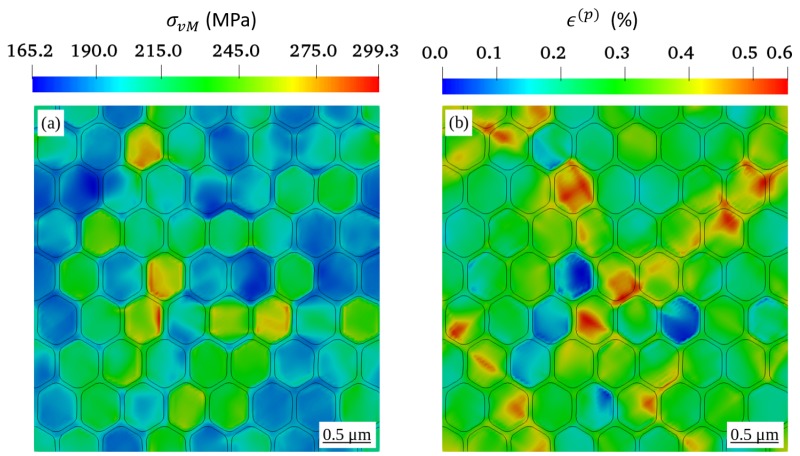
Distribution of (**a**) equivalent stress and (**b**) equivalent plastic strain corresponding to the onset of plastic deformation, which is defined here by a global plastic strain of 0.2%.

The distribution of equivalent stress and strain in the RVEs with a grain diameter of 0.4 μm, corresponding to the green colored arrow pointing to the yield point in Figure 1b, is shown in Figure 2a and Figure 2b, respectively. Because this stage of deformation appears at the onset of plasticity, a certain degree of shear band is observable, and the stress concentration along grain boundaries is not pronounced. The global values of dislocation densities ${\bar{\rho }}_{\mathrm{total}}$, ${\bar{\rho }}_{\mathrm{SSD}}$ and ${\bar{\rho }}_{\mathrm{GND}}$ are evaluated by taking the volume average of the local quantities. Each simulation is run using OpenMP algorithm. The simulations of RVEs with the grain size of 16 μm are run on 8 cores and have taken approximately 18 h. Other simulations of RVEs with grain sizes of 1.6, 0.8, and 0.4 μm, are run on 16 cores and have taken roughly 7, 14.5, and 27 h respectively.

## 4. Results and Discussion

In order to investigate the influence of the grain size, first of all we evaluated the distributions of $\rho_{\mathrm{GND}}$, $\rho_{\mathrm{SSD}}$ and $\rho_{\mathrm{total}}$ in all RVEs at 5% total strain, and then we applied the volume averaged homogenization scheme to investigate the evolution of these quantities with respect to the plastic deformation. After that, we also analyzed the global flow stress ${\bar{\sigma }}_{\mathrm{vM}}$, ${\bar{\rho }}_{\mathrm{GND}}$, ${\bar{\rho }}_{\mathrm{SSD}}$ and ${\bar{\rho }}_{\mathrm{total}}$ from all RVEs at the onset of plasticity and at the total strain of 5.0%. Finally, the sensitivity of the selected material parameters is studied and reported.

### 4.1. Effect of the Grain Size on the Distribution of Dislocation Density

**Figure 3:**
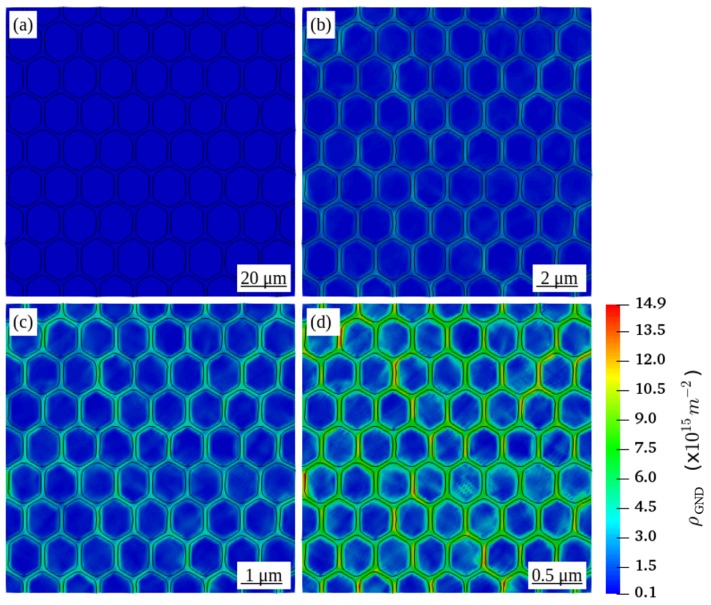
Distribution of geometrically necessary dislocation density $\rho_{\mathrm{GND}}$ in the deformed RVEs with a grain diameter D of (**a**) 16 μm (**b**) 1.6 μm (**c**) 0.8 μm (**d**) 0.4 μm at a global plastic strain of 5%.

Figure 3 shows the distribution of $\rho_{\mathrm{GND}}$ in all RVEs at a global plastic strain of 5%. Comparing to RVEs with a smaller grain diameter, the distribution of $\rho_{\mathrm{GND}}$ in the RVE in Figure 3d with a grain diameter of 16 μm is rather small and negligible. By decreasing the grain diameter, $\rho_{\mathrm{GND}}$ increases and tends to concentrate along the grain boundaries. Such observed concentration of $\rho_{\mathrm{GND}}$ along the grain boundaries is consistent with the large strain gradients in these regions of strain incompatibility between neighboring grains.

**Figure 4:**
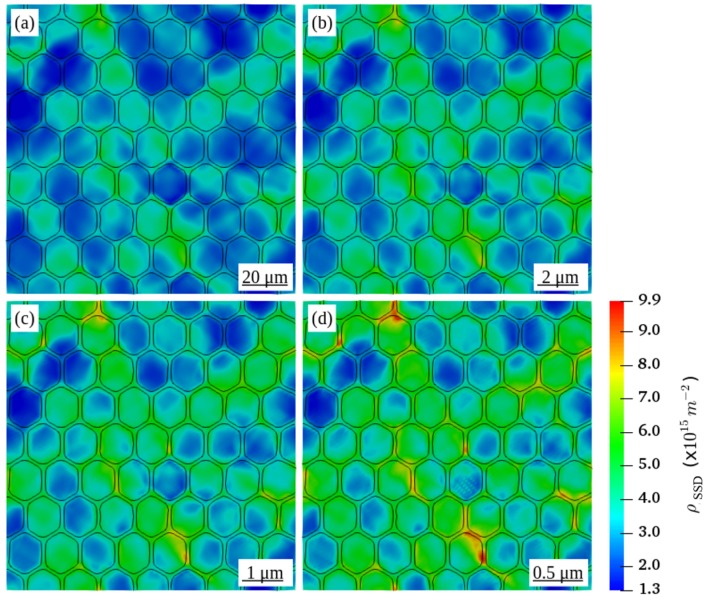
Distribution of statistically stored dislocation density $\rho_{\mathrm{SSD}}$ in the deformed RVEs with the grain diameter D of (**a**) 16 μm (**b**) 1.6 μm (**c**) 0.8 μm (**d**) 0.4 μm at a global plastic strain of 5%.

With respect to the modified form of the Kocks-Mecking law (Equation (11)), the evolution of SSD ${\dot{\rho }}_{\mathrm{SSD}}^{s}$ depends directly upon the $\rho_{\mathrm{GND}}^{s}$. Therefore, the distribution of the $\rho_{\mathrm{SSD}}$ in all RVEs as illustrated in Figure 4, shows that $\rho_{\mathrm{SSD}}$ also increases with decreasing grain size. However, comparing to Figure 3, the effect of the grain size is much less prominent. As the plastic deformation progresses, storage and annihilation of SSD compete with each other to maintain a state of dynamic equilibrium. In addition, the $\rho_{\mathrm{SSD}}$ distribution in all deformed RVEs shows patterns similar to those of the distribution of the equivalent plastic strain as shown in Figure 2, which represents, however, the local plastic strain at a global value of 0.2%.

**Figure 5:**
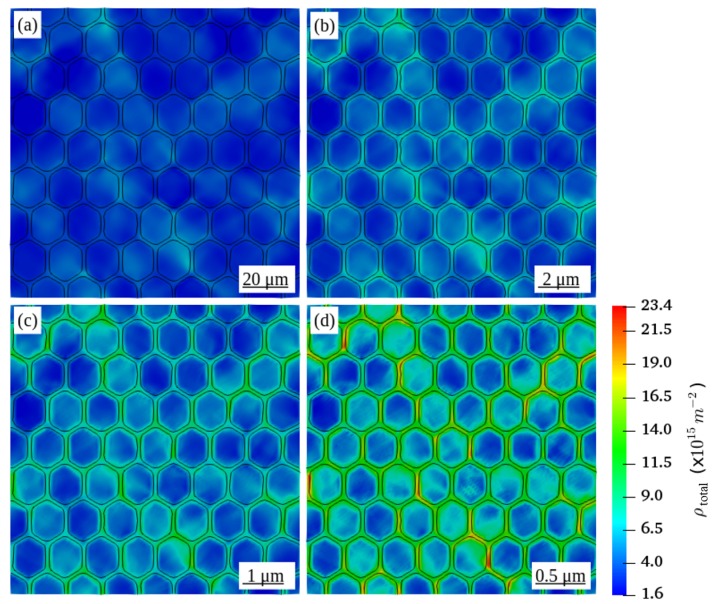
Distribution of total dislocation density $\rho_{\mathrm{total}}$ in the deformed RVEs with a grain diameter D of (**a**) 16 μm (**b**) 1.6 μm (**c**) 0.8 μm (**d**) 0.4 μm at a global plastic strain of 5%.

The total dislocation density distribution in the deformed RVEs at a stage of 5% total strain is shown in Figure 5. Because $\rho_{\mathrm{GND}}$ is much smaller than $\rho_{\mathrm{SSD}}$, the pattern of the distribution of $\rho_{\mathrm{total}}$ for the RVE with a large grain diameter of 16 μm in Figure 5a is similar to the $\rho_{\mathrm{SSD}}$ distribution in the same RVE, as shown in Figure 4a. By decreasing the grain diameter, the pattern of the total dislocation density distribution exhibits an equivalent combination of $\rho_{\mathrm{SSD}}$ and $\rho_{\mathrm{GND}}$ distributions, where both shear bands and localized dislocation density can be observed at grain boundaries.

**Figure 6:**
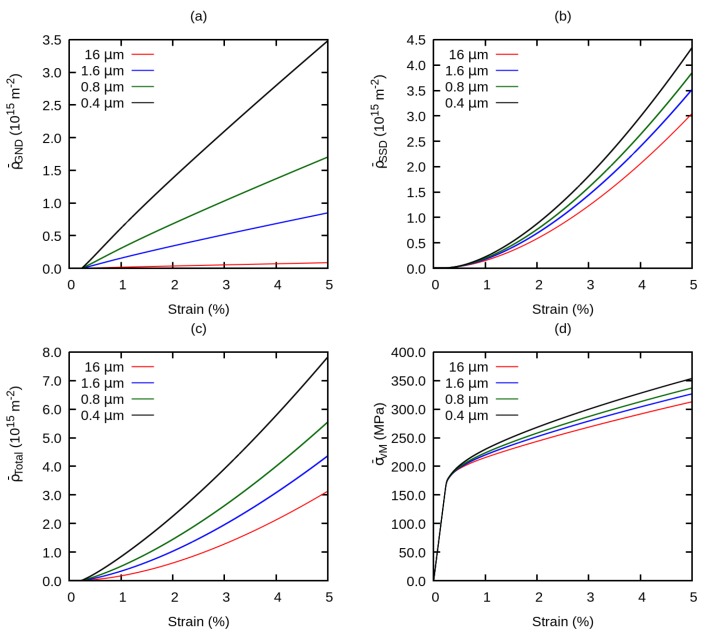
Effect of grain size on the evolution of the global (**a**) geometrically necessary dislocation density, (**b**) statistically stored dislocation density, (**c**) total dislocation density, (**d**) flow stress.

To investigate the contribution of grain size on the evolution of dislocation densities and on the hardening behavior of the material, global dislocation densities ${\bar{\rho }}_{\mathrm{GND}}$, ${\bar{\rho }}_{\mathrm{SSD}}$, ${\bar{\rho }}_{\mathrm{total}}$ and flow stress ${\bar{\sigma }}_{\mathrm{vM}}$ have been plotted versus the total strain in Figure 6. From these curves, all dislocation densities start to increase sharply after a total strain of approximately 0.3%.

**Figure 7:**
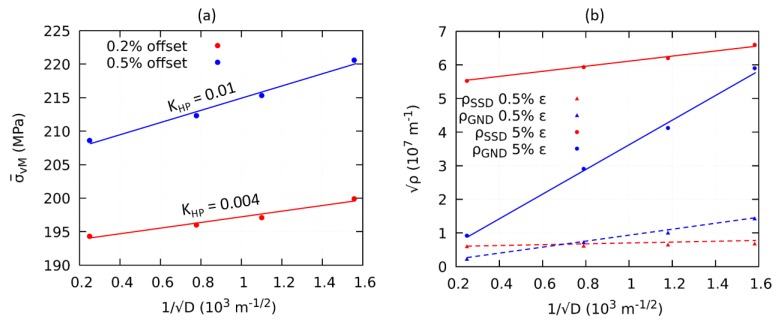
(**a**) Hall-Petch coefficient calculated at an offset of 0.2% and 0.5% of total strain, (**b**) evolution of $\rho_{\mathrm{SSD}}$ and $\rho_{\mathrm{GND}}$ at 0.5% and 5% of total strain with variation in grain size.

In the next step, the influence of grain size on dislocation densities and on the yield stress has been investigated at the onset of plasticity. Firstly, the yield stress measures taken at an offset of 0.2% and 0.5% strain are plotted against the inverse square root of the grain diameter as shown in Figure 7a. The fitted trend lines indicate that the yield stress increased linearly with respect to the inverse square root of the grain diameter and hence followed the Hall-Petch relationship.

To understand the contribution of dislocation densities on grain boundary strengthening, the ${\bar{\rho }}_{\mathrm{SSD}}$ and ${\bar{\rho }}_{\mathrm{GND}}$ at a total strain of 0.5% and 5% are evaluated from all of the RVEs and plotted against the inverse square root of the grain diameter as shown in Figure 7b. Comparing the global plastic strains of 5.0% with 0.5%, ${\bar{\rho }}_{\mathrm{SSD}}$ and ${\bar{\rho }}_{\mathrm{GND}}$ rose in all simulations. The strain gradient or grain size affected the global ${\bar{\rho }}_{SSD}$ negligibly at the small plastic strain, but the effect got prominent at the higher plastic strain, which is indicated by a small increase in the slope of the linear regression i.e., solid red line. To understand this observation, we refer to the evolution of SSD (${\dot{\rho }}_{\mathrm{SSD}}$) given in Equation (11), which involves GND ($\rho_{\mathrm{GND}}$). For the case of $\rho_{\mathrm{GND}}$, the influence of the grain size is strong at smaller plastic strain and stronger at larger plastic strain.

The value of the Hall-Petch coefficient evaluated at 0.2% offset is 0.004 MPa m${}^{1/2}$, which is much lower as compared to the experimental findings reported in [5,9]. It is commonly observed in experiments [5,7,10] that a decrease in grain size leads to an increase in initial yield strength. This behavior is not captured by our modelbecause our formulation follows the Nix/Gao-type theory as explained in [47], which describes a prominent change in hardening rate with decreasing microstructural length scale, while the influence of the length scale on initial yield stress is negligible. A similar observation can be made in Figure 6d, in which hardening rate increases but the initial yield stress does not increase significantly with decreasing grain size. In other work [13], it has been explained in terms of dislocation density concentration in the vicinity of grain boundaries. El-Awady [48] has discussed rigorously the dependence of initial yielding and initial dislocation density on grain size. However, the strain gradient plasticity models, which are based on the classical Kocks-Mecking dislocation evolution law, are not able to capture this behavior without the introduction of suitable prior adjustments. Cheong et al. [8] have employed a grain size dependent initial SSD for different RVEs and [49] have assumed specific storage of GND at grain boundaries prior to plastic deformation and found a dependency of yielding stress on grain size with an exponent of −1.5. Our results follow qualitatively the trends of flow curves reported by [6]. One observation shows that the value of Hall-Petch coefficient increases slightly with progressing plastic deformation, which is consistent with the experimental results reported by [8,10,50]. suggestions

The governing mechanism of strengthening does not only depend on the stored dislocations but also on their annihilation. Therefore, the next section of this work aims at investigating the influence of material parameters that control the initial state and influence of evolution of dislocation densities on the deformation mechanism.

### 4.2. Averaged Stress and Dislocation Density Under the Influence of Model Parameters

**Figure 8:**
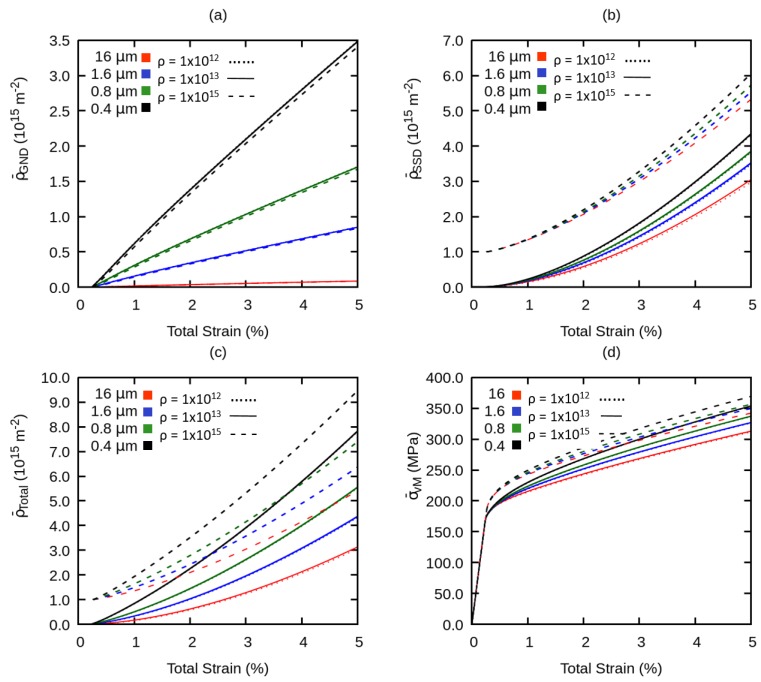
Effect of variation of initial total dislocation density on the global (**a**) geometrically necessary dislocation density, (**b**) statistically stored dislocation density, and (**c**) total dislocation density (**d**) flow stress.

Firstly, we have studied the influence of the initial total dislocation density $\rho_{\mathrm{total}(i)}$ on the grain size effect. The value of the initial total dislocation density is varied from $10^{12}$ to $10^{15}$$m^{-2}$, whereas other parameters are set according to Table 2. The higher value of initial total dislocation density corresponds to an unannealed material configuration whereas lower initial dislocation density mimics an annealed microstructure. We have plotted the evolution of ${\bar{\rho }}_{\mathrm{GND}}$, ${\bar{\rho }}_{\mathrm{SSD}}$, ${\bar{\rho }}_{\mathrm{total}}$, and flow stress ${\bar{\sigma }}_{\mathrm{vM}}$ versus the total strain resulting from the simulations as shown in Figure 8.

Since $\rho_{\mathrm{total}(i)}$ is assumed to be equivalent to $\rho_{\mathrm{SSD}}$, an increase in $\rho_{\mathrm{total}(i)}$ results in an increasing ${\bar{\rho }}_{\mathrm{SSD}}$, but it does not lead to any significant change in ${\bar{\rho }}_{\mathrm{GND}}$. Furthermore, a larger value of $\rho_{\mathrm{total}(i)}$ suppresses minimally the influence of grain size on the evolution of ${\bar{\rho }}_{\mathrm{SSD}}$. This results into an increase of yield stress for all RVEs, but the effect of the grain size on the hardening behavior diminishes as shown in Figure 8d. This can be correlated to the experimental results from [10], that the grain size effect resulting from plastic deformation of unannealed (higher initial dislocation density) materials is weaker as compared to that resulting from plastic deformation of annealed specimens (lower initial dislocation density).

**Figure 9:**
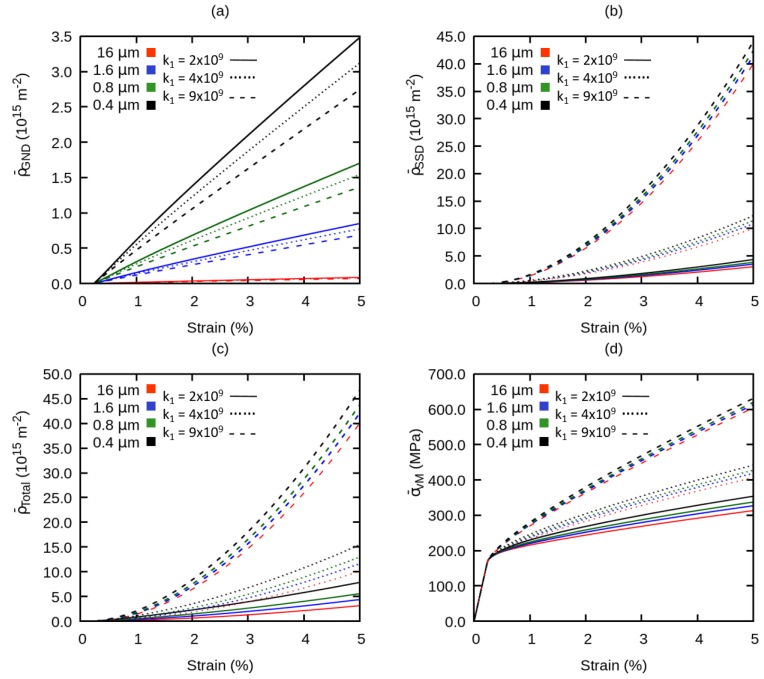
Influence of variation of SSD storage on the global (**a**) geometrically necessary dislocation density, (**b**) statistically stored dislocation density, (**c**) total dislocation density, (**d**) flow stress.

Secondly, to investigate the influence of SSD storage parameter k${}_{1}$, we have compared simulations using parameters from Table 2 with simulations with k${}_{1}$ of 4 × 10${}^{9}$ and 9 × 10${}^{9}$, and the results are shown in Figure 9. In general, a larger value of k${}_{1}$ elevates the evolution of ${\bar{\rho }}_{\mathrm{SSD}}$ significantly but suppresses the evolution of ${\bar{\rho }}_{\mathrm{GND}}$. Consequently, by increasing k${}_{1}$, ${\bar{\rho }}_{\mathrm{total}}$ increases non-linearly and results into a more pronounced hardening behavior. However, because the contribution of ${\bar{\rho }}_{\mathrm{GND}}$ is suppressed, the influence of the grain size on the hardening behavior is minimized.

**Figure 10:**
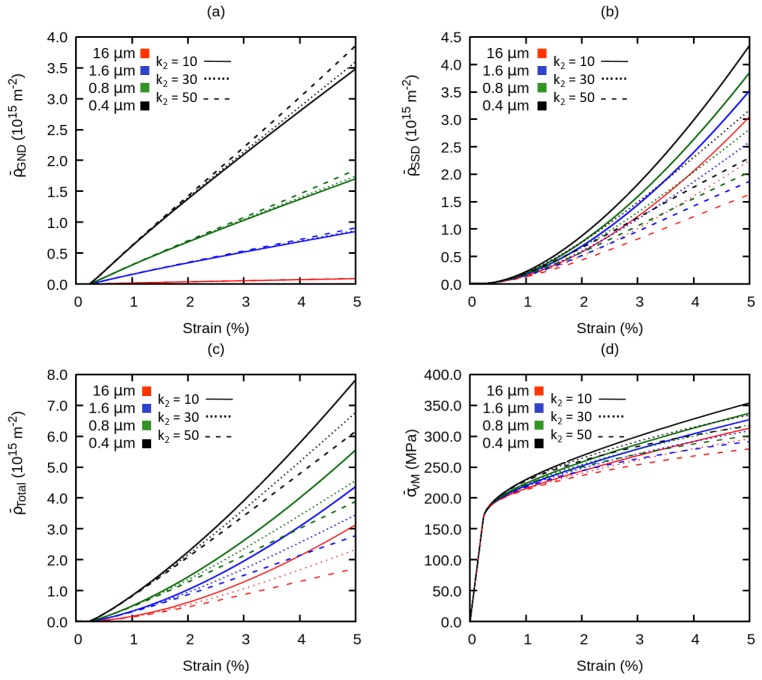
Effect of variation of dislocation annihilation on the global (**a**) geometrically necessary dislocation density, (**b**) statistically stored dislocation density, (**c**) total dislocation density, (**d**) flow stress.

Thirdly, the effect of SSD annihilation parameter k${}_{2}$ on the evolution of global dislocation densities and flow stress is evaluated as plotted in Figure 10. We have increased the magnitude of k${}_{2}$ to 30 and 50. With a lower value of k${}_{2}$, lesser SSD annihilate so the rate of storage of ${\bar{\rho }}_{\mathrm{SSD}}$ is higher. The opposite of this happens with a larger k${}_{2}$, which also promotes the influence of the grain size on ${\bar{\rho }}_{\mathrm{GND}}$ by increasing the rate of storage of $\rho_{\mathrm{GND}}$ as observable in Figure 10a. This effect is, however, of minor importance and it also results into an increase of ${\bar{\rho }}_{\mathrm{total}}$, but at a lower rate with increasing the total strain. As a consequence, a weaker strain hardening behavior is observed but because of the higher storage of the ${\bar{\rho }}_{\mathrm{GND}}$, the influence of the grain size is enhanced.

**Figure 11:**
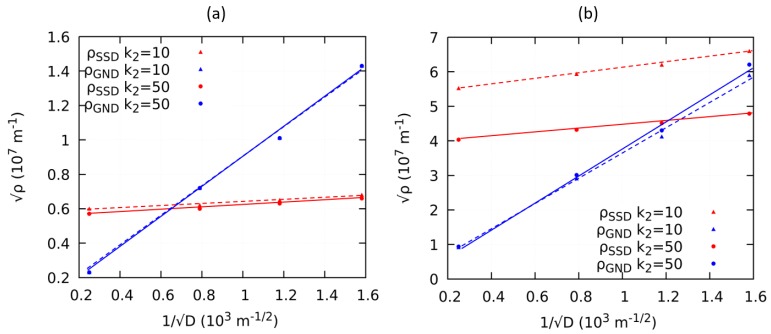
Effect of dislocation annihilation on ${\bar{\rho }}_{\mathrm{SSD}}$ and ${\bar{\rho }}_{\mathrm{GND}}$ at (**a**) 0.5% plastic strain and (**b**) 5% plastic strain.

Finally, the evolution of the global dislocation densities is investigated with respect to a change in the grain size at two particular strain levels with two different SSD annihilation parameters. From the global dislocation densities at 0.5% of the total strain i.e., at the onset of plasticity as shown in Figure 11a, ${\bar{\rho }}_{\mathrm{GND}}$ becomes larger and surpasses ${\bar{\rho }}_{\mathrm{SSD}}$, The ${\bar{\rho }}_{\mathrm{SSD}}$ does not increase significantly with a decreasing grain size at this strain. This means that the contribution of GND to the onset of plasticity is higher and overcomes SSD at smaller grain sizes. k${}_{2}$ does not, however, affect the evolution of the dislocation densities because the material is still at the early stage of deformation. It is also clear that the chosen values 10 and 50 of k${}_{2}$ have a negligible effect on the behavior of dislocation densities. For the case of a larger plastic strain of 5.0% as plotted in Figure 11b, the effect of k${}_{2}$ is much more significant. With the relatively lower k${}_{2}$ of10, annihilation is not pronounced and resulted in an increase of the ${\bar{\rho }}_{\mathrm{SSD}}$. At 5.0% of plastic strain, ${\bar{\rho }}_{\mathrm{GND}}$ does not exceed ${\bar{\rho }}_{\mathrm{SSD}}$. However, by increasing the value of k${}_{2}$ to 50, the evolution of SSD is more suppressed, therefore ${\bar{\rho }}_{\mathrm{SSD}}$ becomes smaller than ${\bar{\rho }}_{\mathrm{GND}}$ at a smaller grain size. This demonstrates that at a larger applied total strain, the dominating type of dislocations during plasticdeformation strongly depended on the values of of k${}_{1}$ and k${}_{2}$.

## 5. Conclusions

In this work, we have implemented dislocation based strain-gradient crystal plasticity into a multi-phase-field framework and investigated the grain size effect together with the contribution of statistically stored dislocation density (SSD) and geometrically necessary dislocation density (GND). Thus, this model is able to predict the behavior of materials in response to applied mechanical loads and describe the changes in mechanical behavior in relation to dislocation densities. The strain-gradient-based nature of the model allows us to analyze the influence of grain size on the strength of a polycrystal. The results are obtained through a series of quasi-2D simulations under different conditions imposed on RVEs with different grain sizes, and the results correlate with the literature. In summary, we can conclude that:

- Our work shows that by applying a dislocation-based strain gradient crystal plasticity model, we can capture many aspects of grain boundary strengthening as it is observed in experiments. This conforms to the Hall-Petch model in which the introduction of special properties for grain boundaries is not necessary.
- The model introduced in our work is capable of recapturing the Hall-Petch relation with an exponent of −0.5 for the grain size dependence. Furthermore our model is consistent with the experimental observations of the evolution of the Hall-Petch coefficient with progressing plastic deformation and the initial state of the material with respect to dislocation density.
- The value of the Hall-Petch coefficient predicted by our model is significantly smaller than those observed through experiments and the strain gradient plasticity is unable to explain the grain boundary strengthening at the onset of plastic yielding. This has been discussed in light of the initial state of the material in particular with respect to the initial GND density prior to mechanical testing.

## Figures and Tables

**Table 1:** Crystallographic vectors.

Slip System	Plane Normal	Slip Direction	Line Direction
s	n	d	l
1	[111]	[1$\bar{1}$0]	[11$\bar{2}$]
2	[111]	[10$\bar{1}$]	[$\bar{1}$2$\bar{1}$]
3	[111]	[01$\bar{1}$]	[$\bar{2}$11]
4	[$\bar{1}$11]	[$\bar{1}\bar{1}$0]	[1$\bar{1}$2]
5	[$\bar{1}$11]	[101]	[12$\bar{1}$]
6	[$\bar{1}$11]	[0$\bar{1}$1]	[211]
7	[1$\bar{1}$1]	[110]	[$\bar{1}$12]
8	[1$\bar{1}$1]	[10$\bar{1}$]	[121]
9	[1$\bar{1}$1]	[0$\bar{1}\bar{1}$]	[21$\bar{1}$]
10	[1$1\bar{1}$]	[1$\bar{1}$0]	[$\bar{1}\bar{1}\bar{2}$]
11	[11$\bar{1}$]	[101]	[1$\bar{2}\bar{1}$]
12	[11$\bar{1}$]	[011]	[2$\bar{1}$1]

**Table 2:** Parameters used in this study.

Parameters	Symbol	Value	Unit	Ref.
Anisotropic elastic constant	**C** ${}_{11}$	108.2	GPa	[43]
Anisotropic elastic constant	**C** ${}_{12}$	61.3	GPa	[43]
Shear Modulus	**C**${}_{44}$ = G	28.5	GPa	[43]
Strain rate sensitivity	m	0.025	-	
Lattice friction stress	$\tau_{o}$	80	MPa	
SSD storage parameter	k${}_{1}$	2 × $10^{9}$	-	[40]
SSD annihilation parameter	k${}_{2}$	10	-	[40]
Initial total dislocation density	$\rho_{\mathrm{total}(i)}$	1 × $10^{13}$	m${}^{-2}$	
Geometrical factor for flow stress	c${}_{1}$	0.3	-	[20,44]
Referential dislocation velocity	$\nu_{0}$	1 × $10^{-3}$	ms${}^{-1}$	
Interfacial energy	$\sigma_{\alpha \beta }$	0.24	Jm${}^{-2}$	[28]
Spacediscretization	Δx	0.1	μm	
Timediscretization	Δt	1	μs	
Interfacial width	η	4.5	Δx	
Domain size	Ω	128 × 128	Δx	
Length of Burger’s vector	b	0.286	nm	

## Nomenclature

CP	Crystal plasticity
MPF	Multi phase field
GND	Geometrically necessary dislocations
SSD	Statistically stored dislocations
RVE	Representative volume element
$\tau^{s}$	Resolved shear stress on s slip system
$\tau_{c}^{s}$	Critical resolved shear stress on s slip system
$\nu^{s}$	Dislocation velocity on s slip system
$\nu_{0}$	Initial dislocation velocity
$\epsilon^{\left(p\right)}$	Equivalent plastic strain
$\dot{\gamma }$	Shear strain rate
c${}_{1}$	Geometrical constant
G	Shear modulus
s	Arbitrary slip system
Ω	Domain/system size
ϕ	Order parameter/phase field parameter
α	Arbitrary name for a phase phase/grain
F	Total free energy of the system
$f^{\mathrm{int}}$	Interfacial free energy
$f^{\mathrm{el}}$	Elastic or mechanical free energy
N	Total number of slip systems or phases
η	Interfacial width
$\rho_{\mathrm{total}}$	Density of dislocations
$\rho_{\mathrm{SSD}}$	Density of SSD
$\rho_{\mathrm{GND}}$	Density of GND
k${}_{1}$	SSD storage parameter
k${}_{2}$	SSD annihilation parameter
m	Strain rate sensitivity parameter
$\sigma_{\alpha \beta }$	Interfacial energy between arbitrary phase or grain α and β
$P^{s}$	Symmetric part of Schmidt tensor on slip system s
$\sigma_{\mathrm{vM}}$	von Mises equivalent stress
ϵ	Total strain tensor
$\epsilon^{\mathrm{el}}$	Elastic strain tensor
$\epsilon^{*}$	Eigen strain tensor
$\epsilon^{\left(p\right)}$	Plastic strain tensor
$\sigma_{\mathrm{ij}}$	Stress tensor
Λ	Nye’s dislocation tensor
C	Stiffness tensor
F	Deformation gradient
b	Burgers vector
d	Slip direction vector
l	Slip plane tangent vector
n	Slip plane normal vector
